# Optical Coherence Tomography Angiography in the Assessment of Vulvar Lichen Sclerosus Vascularity and Epithelial Thickness In Vivo

**DOI:** 10.1002/tbio.70000

**Published:** 2025-04-24

**Authors:** Raksha Sreeramachandra Murthy, Christina N. Kraus, Felicia Lane, Zhongping Chen

**Affiliations:** 1 Department of Biomedical Engineering, University of California, Irvine, California, USA; 2 Beckman Laser Institute, University of California, Irvine, California, USA; 3 Department of Dermatology, University of California, Irvine, California, USA; 4 Department of Gynecology, University of California, Irvine, California, USA

**Keywords:** dermatology, differentiated vulvar intraepithelial neoplasia, OCT angiography, oncology, optical coherence tomography, squamous cell carcinoma, vulvar lichen sclerosus

## Abstract

Vulvar lichen sclerosus is a chronic inflammatory skin condition that leads to scarring and an increased risk of squamous cell carcinoma. It presents clinically as atrophic white patches or plaques, often with associated fissures, erosions, hyperkeratosis, purpura or ecchymoses. The chronic inflammation in vulvar lichen sclerosus leads to permanent scarring, resulting in pain syndromes and genitourinary complications. While diagnosis is often made clinically, skin biopsies are considered the gold standard for diagnosis. Additionally, biopsies are often required throughout the course of the disease to monitor for the development of malignancy. Thus, there is a need for noninvasive, high-sensitivity, real-time imaging to evaluate vulvar lichen sclerosus changes. This study presents a proof-of-concept evaluation of a 1.7-μm optical coherence tomography (OCT)/OCT angiography (OCTA) system with enhanced penetration depth and high resolution for characterizing the structural and microvascular features of VLS. The primary objective was to evaluate the feasibility of using this imaging technology to quantitatively measure vulvar epithelial thickness and vascular changes across different anatomical sites (labia majora, labia minora, and interlabial sulci) in both VLS patients and healthy controls. By leveraging the increased penetration depth of the 1.7-μm OCT system, we aimed to provide a deeper understanding of VLS-associated tissue alterations and explore its potential as a non-invasive alternative to biopsies for disease assessment and monitoring.

## 1 | Introduction

Lichen sclerosus (LS) is a chronic inflammatory skin condition that has a predilection for the anogenital skin in women [1]. While the true prevalence of vulvar LS (VLS) is unknown, as it is likely underdiagnosed and underreported, studies have estimated that VLS affects up to 3% of postmenopausal women [2]. Overall, this is an underserved condition, and the related delay in diagnosis can have a profound burden on patients’ quality of life and health outcomes, leading to irreversible scarring, infection, vulvar architectural distortion, sexual dysfunction, genitourinary complications, itch, and pain syndromes [2]. Around half of all cases of vulvar cancer emerge in the presence of VLS [3]. Vulvar LS can be challenging to diagnose clinically as skin findings may be subtle initially [4] Additionally, in the setting of longstanding VLS, chronic scarring and epidermal changes (e.g., lichenification or hyperkeratosis) can make it challenging to monitor for squamous cell carcinoma (SCC) or its precursor, differentiated vulvar intraepithelial neoplasia (dVIN), within longstanding LS lesions [5]. Thus, biopsies are often utilized for diagnosis of VLS and for monitoring to evaluate lesions of concern for malignant change.

Histologically, the classic presentations of VLS consist of collagen homogenization in the dermis, epidermis changes (including atrophic and hypertrophic changes), as well as vascular changes, with dilated vessels early in the disease course and later with sclerosis of vessels [6]. However, histological variants including non-sclerotic VLS have recently been reported [7] and studies have found histological and clinical discordance in the diagnosis of VLS due to the heterogeneity of this disease [8]. Such limitations in the interpretation of biopsy specimens may lead to misdiagnosis and insufficient monitoring, while also contributing to patient hesitancy to undergo additional biopsies in the future when prior biopsies were unrevealing. Finally, vulvar biopsies present inherent drawbacks, characterized by their invasiveness as well as technical intricacies. These procedures may contribute to patient discomfort intra- and post-procedure and have the potential to exacerbate disease activity through koebnerization or scar formation [9]. Thus, there is increased demand for noninvasive, real-time imaging methods with high sensitivity to diagnose and monitor VLS.

Optical coherence tomography (OCT) is a novel imaging modality for skin disease screening and diagnosis and has been developed to improve the diagnostic accuracy of cancer [10–12]. A recent prospective study highlighted the utility of a commercial OCT for the diagnosis of vulvar disease, in which benign vulvar neoplasms and inflammatory skin conditions (including VLS) were detected with a 1.3-μm OCT and compared to histopathology, with a sensitivity of 83%, specificity of 57%, and accuracy of 78% [13]. Previous OCT studies of VLS faced challenges in epidermal thickness quantification, with limitations arising from multiple factors. While some studies identified technical constraints related to vulvar anatomical irregularity or image resolution [8, 13], recent work has demonstrated that in moderate-to-severe VLS lesions, the altered optical properties of sclerotic dermal collagen and epidermal hyperkeratosis fundamentally disrupt the contrast between tissue layers, making traditional OCT boundary detection unreliable [14]. Our group recently developed an OCT system utilizing a 1.7-μm wavelength source, enabling in vivo characterization of vaginal tissue, cardiovascular tissue, and skin tissue. This system offers a 25% improvement in penetration depth compared to conventional OCT systems while maintaining high resolution [10, 15, 16]. We have implemented a similar setup for the characterization of VLS. Through high-resolution 1.7-μm OCT/OCTA imaging, we analyze the dynamics of the vulvar epithelium, blood vessel density, and diameter at different vulvar sites in patients with VLS. Our OCT algorithm also addresses the limitations of existing studies by enabling the calculation of epidermal thickness in the uneven vulvar area [8]. The results of this pilot study will serve as a pioneering tool for potential diagnosis and therapeutic monitoring. Ultimately, our goal is to utilize these findings to develop a standardized and reproducible measure that can be assessed through OCT/OCTA and would have the potential to be utilized in studies evaluating disease activity and treatment response, moving toward developing standardized outcomes measures for future clinical trials for VLS.

## 2 | Materials and Methods

### 2.1 | System Set-Up

**FIGURE 1 |:**
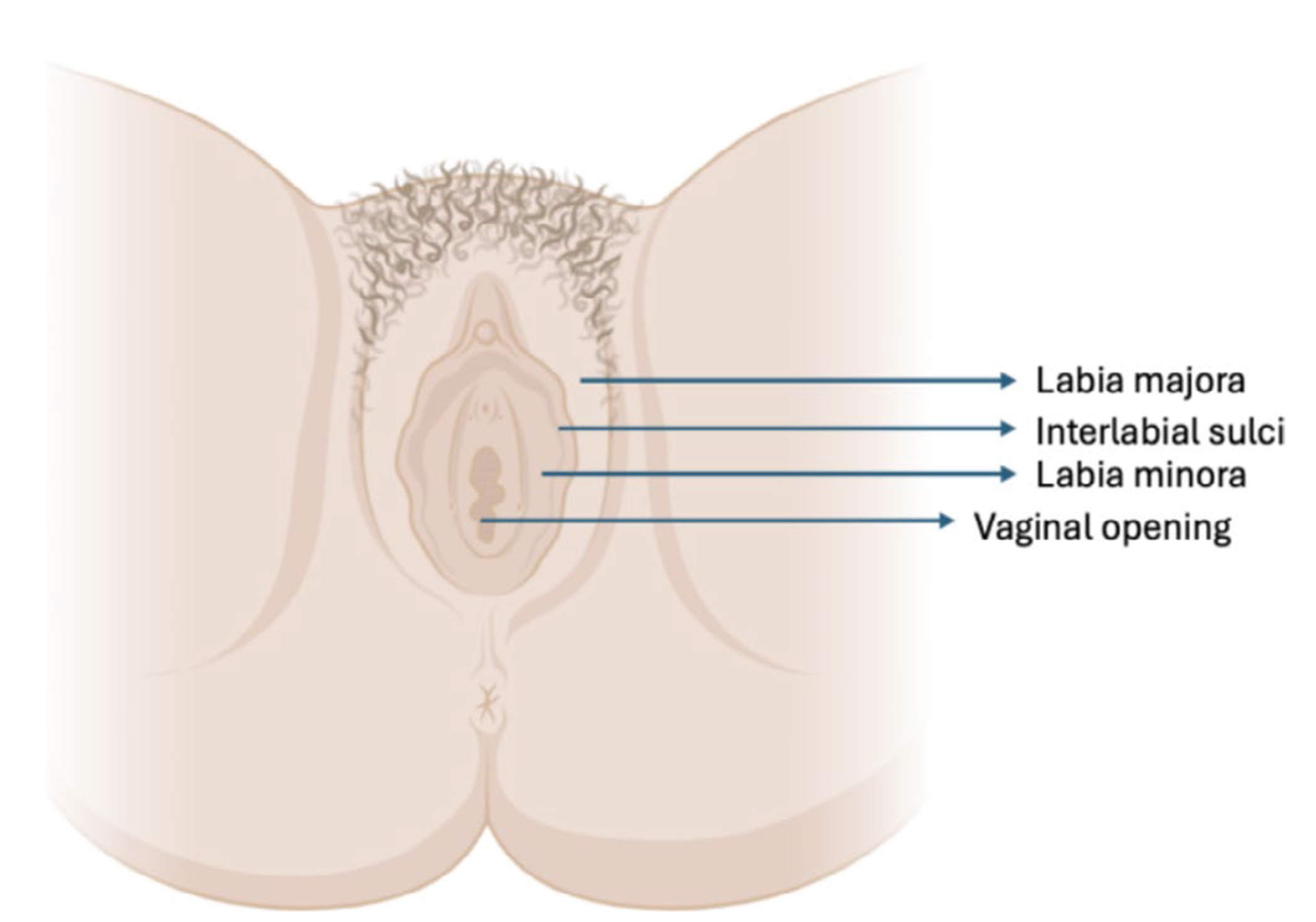
Anatomy of the vulva (created with BioRender.com).

The system consists of a 1.7-μm Swept-Source laser as shown in Figure 1. The output energy of the laser is 22 mW. It is split by a 90:10 coupler. The sample arm is supplied by 90% of the power and 10% is supplied to the reference arm. The system is equipped with a lambda-trigger for increased phase stability. Two circulators with a center wavelength of 1650 nm are used to separate the illumination light and back reflected light. The interference signal generated by the sample and reference arm in the 50:50 coupler is detected by the photodetector. The data acquisition software are written in C++, allowing for faster data processing and real-time displaying of images.

**FIGURE 2 |:**
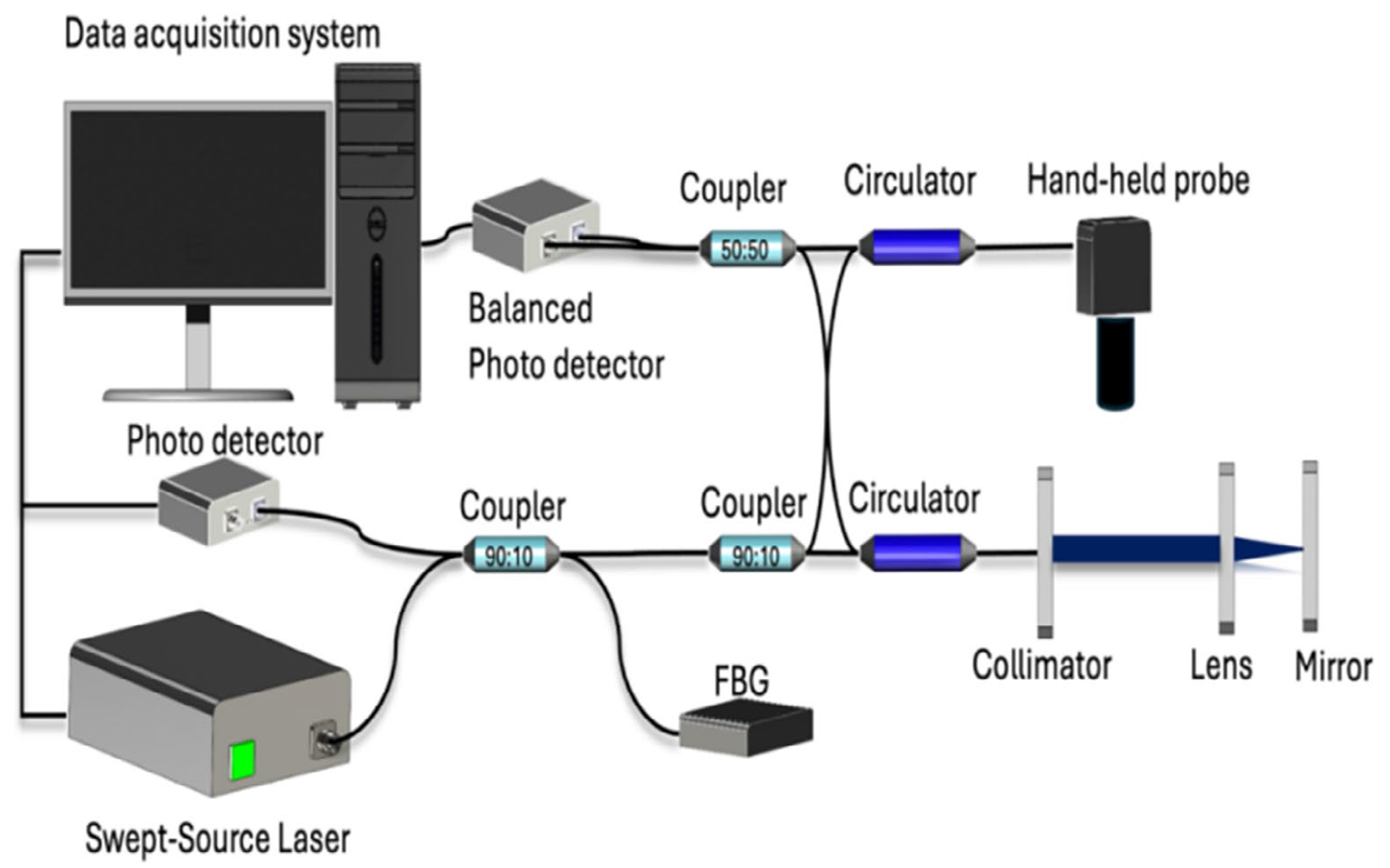
OCT/OCTA schematic.

In the handheld probe for 3D OCT imaging, several components are used to achieve the desired imaging results. These include a dual-axis galvanometer, a scan lens, and a threaded adapter. The dual-axis galvanometer is responsible for scanning the laser beam in two dimensions. The beam is scanned across the 5 mm-by-5 mm target area, allowing for the collection of cross-sectional images. The scan lens is used to focus the laser beam onto the target area. It ensures that the beam is tightly focused and provides the lateral resolution of 40 μm. A threaded adapter is connected to the probe and allows for easy adjustment of the distance between the probe and the imaging target. By rotating the adapter, the depth of focus can be fine-tuned, ensuring that the target area remains within the optimal imaging range. This adjustment helps maintain the desired image quality and focus throughout the scanning process. By combining the dual-axis galvanometer for beam scanning, the scan lens for focusing the beam, and the threaded adapter for depth of focus adjustment, the handheld probe enables 3D OCT imaging with accurate and adjustable imaging capabilities Figure 2.

### 2.2 | Scanning Protocol

OCTA captures a series of consecutive images to detect areas with flow changes. In this study, an interframe scanning protocol is applied to extract vascular information. In the inter-frame imaging protocol, neighboring B-scans are compared to extract vascular information [17]. Six cross-sectional B-scan images are obtained at the same position during image acquisition. This protocol has a longer time interval Δ*T* = 5 ms as it utilizes the slow scan of the scanning apparatus. Intensity-based doppler variance algorithm is applied to obtain doppler OCT images. Doppler OCT images are re-sliced along the depth direction to obtain enface OCTA images. Intensity-based doppler variance (IBDV) is described by the following equation.

(1) $$\sigma^{2}=1-\frac{\overset{J}{\underset{j=1}{\sum }}\left|A_{j,z}\right|\left|A_{j+1,z}\right|}{\overset{J}{\underset{j=1}{\sum }}\left(\frac{{\left|A_{j,z}\right|}^{2}{\left|A_{j+1,z}\right|}^{2}}{2}\right)}$$

where $\left|A_{j,z}\right|$ is the amplitude signal at *j^th^* A-line at a depth of *z*, *J* is the number of averaged A-lines. Since the time interval between neighboring A-lines is longer, the inter frame based IBDV method provides enhanced sensitivity for microvasculature imaging.

### 2.3 | Image Processing

**FIGURE 3 |:**
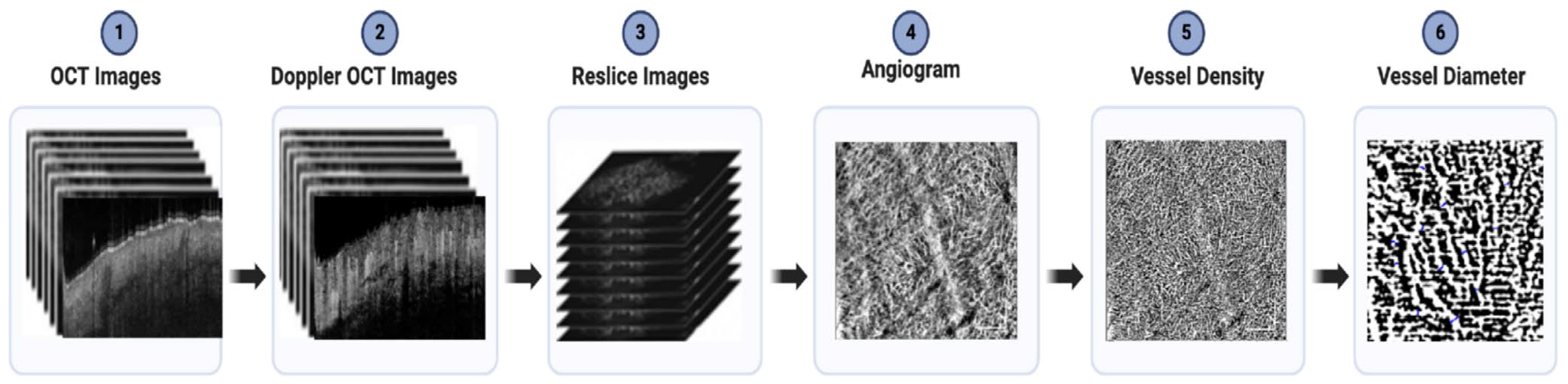
Image Processing steps. This figure illustrates the step-by-step process for analyzing vessel density and diameter in OCT images. (1) Raw OCT images are acquired from tissue samples. (2) Doppler OCT images are generated using an intensity-based doppler variance algorithm. (3) The images are resliced along the depth direction. (4) Angiograms are generated using a 3D maximum intensity projection technique to visualize the vascular network. (5) Vessel density is calculated as the percentage of the area occupied by blood vessels in the labia minora region. (6) Vessel diameter is measured by analyzing the vascular structures at various points in the zoomed-in diagram. The width of individual vessels is measured, quantified, and averaged to provide a comprehensive measurement. Scale bar = 1 mm.

Vessel density and diameter were calculated using MATLAB R2022b (version: 9.13.0 (R2022b)) [18] and ImageJ (version Java 1.8.0_322, Bethesda, MD) [19]. After generating enface OCTA images, several image processing steps were applied to quantify vessel density, vessel diameter, and epidermal thickness as shown in Figure 3. The enface OCTA images were projected along the depth (*z*) axis to generate angiograms, which offer a two-dimensional representation of vascular structures throughout the entire imaging volume. This projection method enhances the visibility of blood vessels and microcirculatory patterns, facilitating the identification and characterization of vascular features. En face OCTA images were binarized using the Mexican Hat filter plugin in ImageJ to enhance vessel contrast and segmentation. To quantify vessel density, we calculated the percentage of vascular structures within a 5 mm × 5 mm region of the Labia Minora. This approach provided a quantitative measure of tissue vascularity. Specifically, we determined the proportion of the defined area that was occupied by blood vessels, yielding a numerical representation of vascular density in the examined tissue. Vessel diameter was quantified by analyzing segmented vascular structures within the angiogram. The width of individual vessels was manually measured at multiple points along their length using the “Analyze and Measure” function in ImageJ to account for variability. These measurements were then averaged to obtain a representative vessel diameter, offering insight into vascular morphology and its role in tissue health and disease progression. VuET was manually measured using ImageJ. Measurements were conducted across different anatomical regions, including the labia majora, interlabial sulci, and labia minora, to evaluate site-specific variations in epidermal thickness.

### 2.4 | In Vivo Imaging

OCT measurements were conducted on a cohort of eight subjects with the objective of measuring alterations in the vasculature and vulvar epithelial thickness (VuET) in VLS. This study was approved by the UC Irvine IRB. Patients ranged from 55 to 80 years of age. All five VLS patients included had a biopsy-proven diagnosis of VLS prior to enrolling in the study and had symptoms of pruritus and clinical signs of active VLS at the time of imaging. The control patients did not have a diagnosis of VLS. Patients with other vulvar dermatoses or evidence of vulvar infection or other vulvar inflammation were excluded. Patients had not applied topical steroids to the area for 2 weeks prior to imaging and were not on any systemic therapies for VLS at the time of imaging. We measured three anatomic locations to include the labia majora, interlabial sulci, and labia minora, as shown in Figure 1. The labia majora consisted of the most external vulvar skin, while the interlabial sulci was the transition between the labia majora and labia minora, and the labia minora consisted of the partially keratinized squamous epithelium that surrounds the vestibule. Quantitative analysis of each vulvar site measured is in Table 1.

## 3 | Results

**FIGURE 4 |:**
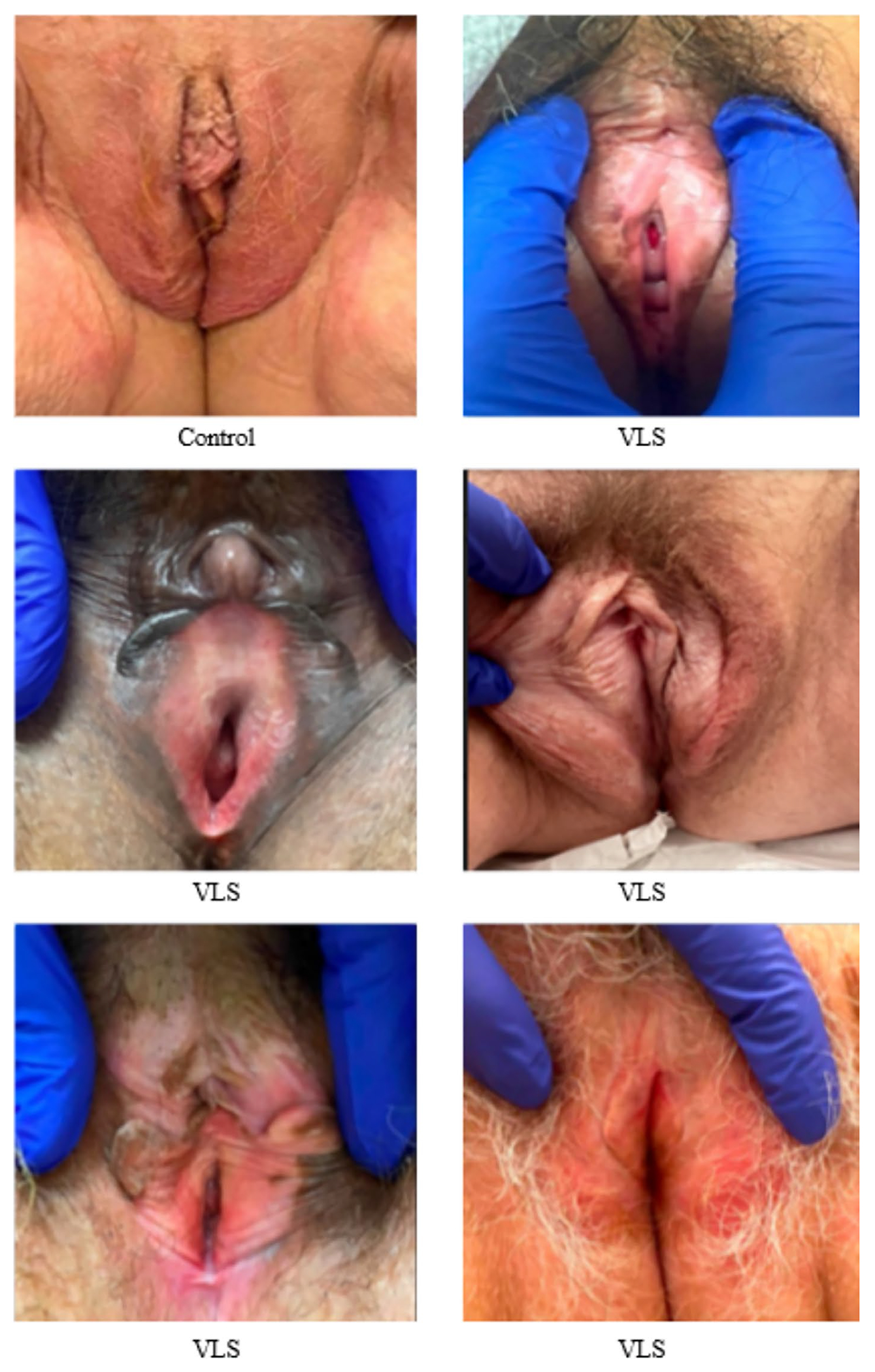
Clinical images of control and VLS subjects.

**FIGURE 5 |:**
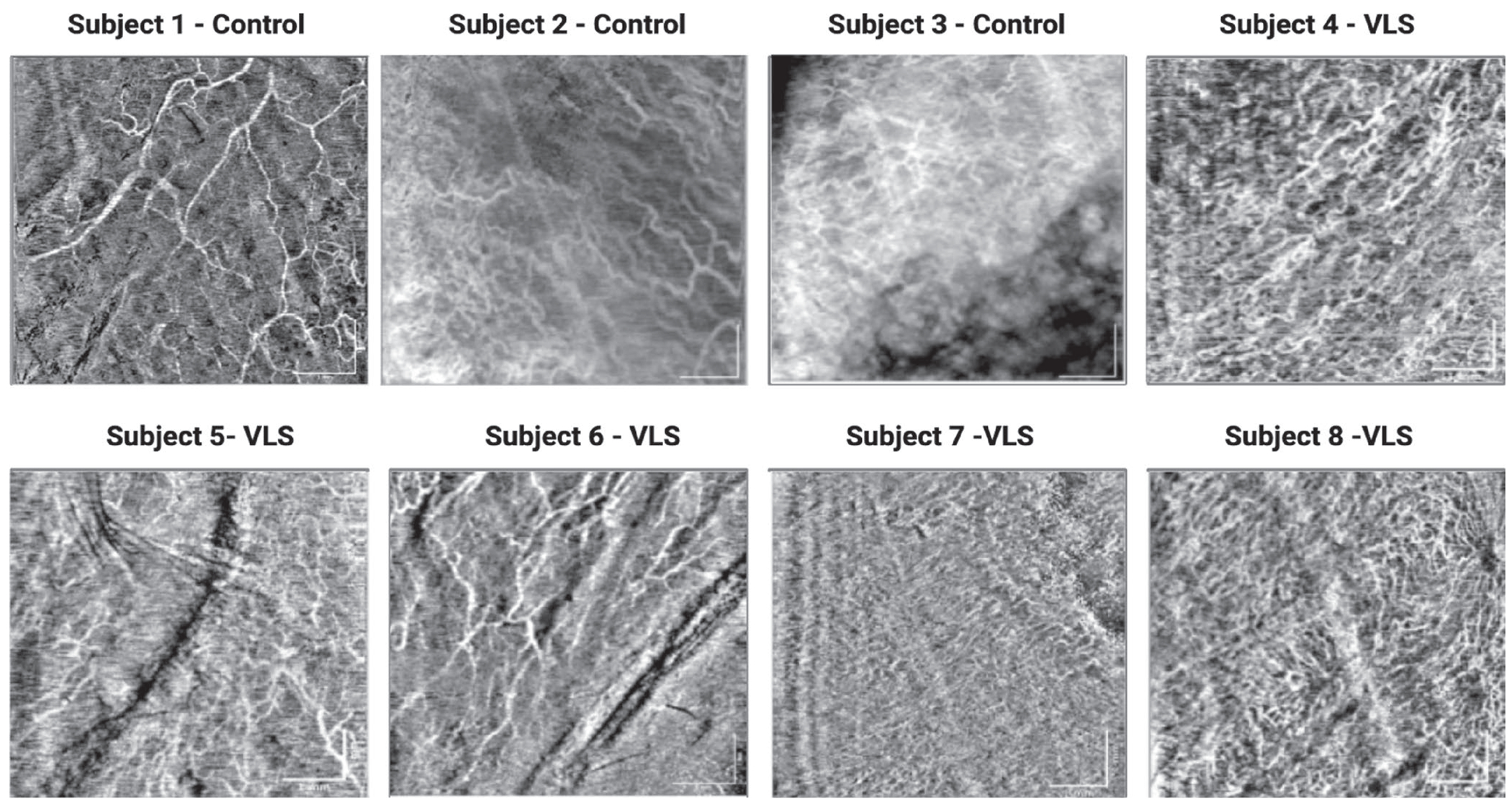
OCT/OCTA images of control and VLS subjects. Scale bar = 1 mm.

**FIGURE 6 |:**
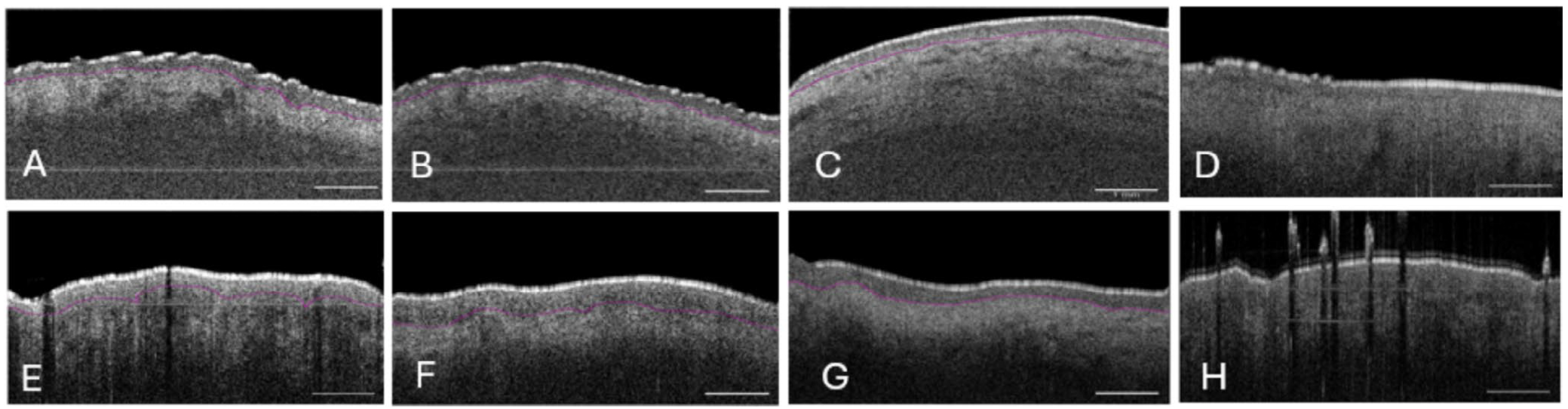
Representative B-scan OCT images of healthy and VLS subjects. (A) Labia majora of a healthy subject. (B) Interlabial sulci of a healthy subject. (C) Labia minora of a healthy subject. (D) Homogenization of the dermis observed in the labia minora of a VLS patient, resulting in reduced optical contrast. (E) Labia majora of a VLS patient. (F) Interlabial sulci of a VLS patient. (G) Labia minora of a VLS patient. (H) Labia majora of a VLS patient, with imaging clarity reduced due to interference from terminal hair. Scale bar = 1 mm.

In all patients, we effectively quantified vessel density (%) (ranging from 32.97% to 45.8%), vessel diameter (ranging from 31.44 to 55.88 μm), and thickness of labia minora epithelium (ranging from 89 to 122 μm). We quantified the epithelial thickness of the labia majora in seven patients, the interlabial sulci in six patients, and the labia minora in eight patients. Figure 4 shows clinical images of six subjects, while Figure 5 presents OCTA images of the labia minora from eight subjects. Overall, we observed a trend of decreasing VuET measurements at the intra-patient level as the epithelium transitioned from keratinized epithelium (labia majora) to modified mucous membranes (interlabial sulci and labia minora) Figure 6.

In VLS cases overall, the vascular structures appeared less well-defined. Our study provides precise measurements of epidermal thickness in distinct anatomical regions of the vulva in VLS patients, with overall changes in the epidermal thickness as the epithelium transitioned from keratinized epithelium (labia majora) to the partially keratinized squamous epithelium (labia minora). While the collection of this pilot data revealed interpatient variability in measurements of VuET, we were able to quantify epithelial thickness and vascularity at an intrapatient level, which differed between sites of vulvar epithelium at different anatomic vulvar sites.

## 4 | Discussion

The non-invasive nature of OCT/OCTA technology renders it indispensable as a method for monitoring tissue changes over time and in response to treatment. These measurements offer valuable insights into the structural characteristics of the vulva.

Vulvar skin is an underserved area of medical research, so there is limited evidence on the use of non-invasive imaging modalities for VLS.

Previous OCT studies of VLS faced challenges in epidermal thickness quantification, with limitations arising from multiple factors. While some studies identified technical constraints related to vulvar anatomical irregularity or image resolution [8, 13], recent work has demonstrated that in moderate-to-severe VLS lesions, the altered optical properties of sclerotic dermal collagen and epidermal hyperkeratosis fundamentally disrupt the contrast between tissue layers, making traditional OCT boundary detection unreliable [14].

In this study, we present a novel 1.7-μm OCT/OCTA system that can effectively assess vulvar VuET and vascularity, as characterized by vessel density and vessel diameter, in vitro with extended depth penetration. Our OCT algorithm overcomes the limitations of existing studies by facilitating the calculation of epidermal thickness in the uneven vulvar area. Further studies are necessary to assess these measures longitudinally at an intra-patient level to evaluate changes in VLS over time and with treatment. VuET and vascularity as assessed by OCT/OCTA are measurements that may have the potential to be assessed over time in select patients to evaluate natural disease course and/or treatment response. If these measurements are reproducible, this system may be a non-invasive method for evaluating tissue changes in VLS.

This was a proof-of-concept pilot study, with eight patients included, five with VLS. In future studies, we aim to apply deep learning algorithms [20, 21] to explore longitudinal measurements over time in patients with VLS to follow the natural disease course, assess changes in measures with the initiation of treatment modalities, and investigate whether this system may be of utility in the early detection of pre-malignant and malignant transformation in VLS. The potential clinical impact of this non-invasive imaging technology on vulvar health and disease is far-reaching, as accurately measuring vulvar changes in vivo has the potential to offer personalized care in monitoring and treating vulvar lichen sclerosus and potentially standardize outcome measurements for vulvar disease.

1.7-μm OCT/OCTA system has numerous potential clinical applications. Currently, pathologic changes of the vulva are identified through physical examination and histologic evaluation. Non-invasive techniques such as ultrasound can be used to evaluate cutaneous pathologies of the vulva [22], but their effectiveness is limited by the technique’s poor axial resolution. OCT offers the ability to detect images on a micrometer scale compared to ultrasound. The main strength of our study is that the 1.7-μm OCT/OCTA system utilized in imaging VLS lesions is developed in-house and provides a 25% greater penetration compared with commercially available systems. This capability allows for visualizing morphology and vasculature in different epithelial sites of the vulva. Finally, the use of multiple measurements at distinct epithelial sites in an individual patient allowed us to evaluate epithelial transitions in the vulvar skin. The main limitations include the inclusion of only eight patients and the absence of histologic correlation with OCT/OCTA measurements. Additionally, in two patients, the dermal-epidermal junction (DEJ) was not clearly distinguishable, raising questions about whether this limitation arises from pathophysiological changes associated with VLS or technical constraints of the imaging system. Since we did not scan patients across a full spectrum of disease severity or perform histological correlation via biopsy, it remains uncertain whether these challenges are inherent to severe VLS pathology or related to optical limitations. Future studies with a larger cohort encompassing varying disease severities will be crucial in better understanding these challenges and determining whether the 1.7-μm OCT system can overcome these limitations through reduced scattering. Furthermore, longitudinal studies tracking changes in optical contrast over time may provide valuable insights into how disease progression influences the ability to delineate epithelial thickness.

## 5 | Conclusion

In summary, we present a proof-of-concept pilot study exploring the potential of a 1.7-μm OCT/OCTA system, which offers a 25% greater penetration compared to commercially available systems, to quantify vulvar epithelial thickness (vuET) and vascularity in VLS. The study also examines variability in these measurements across different anatomical sites of the vulvar epithelium. These findings could be extended to increase our knowledge of vulvar anatomy and epithelial transitions in vulvar skin in both health and disease, with the overall goal of improving patient care and outcomes.

## Figures and Tables

**TABLE 1 |:** Quantitative measurements across distinct vulvar sites.

				Vulvar epithelial thickness (VuET)		
Subject	VLS/control	Vessel density labia minora (%)	Vessel diameter labia minora (μm)	Labia majora (μm)	Interlabial sulci (μm)	Labia minora (μm)
1	Control	32.97	55.88 ± 18.1	317	192	110
2	Control	36.156	53.9 ± 7.39	206	182	144
3	Control	29.21	51.7 ± 9.5	196	155	131
4	VLS	39.82	41.38 ± 13.78	221	—	181
5	VLS	37.09	58.27 ± 14.23	—	—	122
6	VLS	40.56	50.8 ± 16.39	177	147	118
7	VLS	45.8	31.44 ± 8.2	162	147	121
8	VLS	40.32	40.92 ± 10.3	121	63	89

## Data Availability

The data that support the findings of this study are available on request from the corresponding author. The data are not publicly available due to privacy or ethical restrictions.
